# The full-length mitochondrial genome of the Fernholm’s hagfish, *Myxine fernholmi* (Myxini; Myxiniformes; Myxinidae)

**DOI:** 10.1080/23802359.2019.1674731

**Published:** 2019-10-11

**Authors:** Keun-Yong Kim, Hong Keun Park, Seok-Gwan Choi, Yun-Hwan Jung, Dae-Sung Lee, Yun-Sook Kim, Jong Su Yoo, Moongeun Yoon

**Affiliations:** a AquaGenTech Co., Ltd, Busan, Republic of Korea;; b Department of Science, Trine University, Angola, IN, USA;; c Distant Water Fisheries Resources Division, National Institute of Fisheries Science, Busan, Republic of Korea;; d National Marine Biodiversity Institute of Korea, Seocheon, Republic of Korea

**Keywords:** Fernholm’s hagfish, mitochondrial genome, *Myxine fernholmi*, phylogeny

## Abstract

The full-length mitochondrial genome of the Fernholm’s hagfish, *Myxine fernholmi* (Myxini; Myxiniformes; Myxinidae) was analyzed by the primer walking method. Its mitogenome was 18,862 bp in total length and was composed of 13 protein-coding genes, two ribosomal RNA genes, and 22 transfer RNA genes. The gene content and order were congruent with those of typical vertebrates. In the phylogenetic tree, *M. fernholmi* showed the closest relationship to *M. glutinosa* in the same genus and subfamily and well separated from the other hagfish in the subfamily Eptatretinae.

Hagfish along with lampreys are classified as cyclostomes (or jawless fish) and regarded as the most primitive lineages of vertebrates. They have the sisterhood relationship to gnathostomes (or jawed vertebrates) (Takezaki et al. 2003). Fernholm’s hagfish, *Myxine fernholmi* inhabits off southern Chile and the Falkland Islands (Mincarone and Soto 2001). This species is found on upper slopes and at bathyal zones, and only few specimens are recorded by commercial bottom trawlers in bycatch (Wisner and McMillan 1995; Collins et al. 1999). Thus, IUCN Red List evaluates it as Least Concern (Mincarone 2011). In this study, we analyzed the full-length mitochondrial genome (mitogenome) of *M. fernholmi* for the first time and reconstructed the phylogenetic tree to reveal its relationship among hagfish.

A specimen of *M. fernholmi* was caught by an observer as a by-catch of a demersal trawler around the Falklands Islands in 2015 (47°00′60″S, 60°21′00″W) and deposited in the National Marine Biodiversity Institute of Korea (MABIK Lot No. 0016059). Genomic DNA was extracted according to Asahida et al. (1996). Its mitogenome was amplified through two independent and overlapping PCR runs, and the PCR products were sequenced using 29 sequencing primers. The sequence was deposited in the GenBank under the accession number MK950150.

Three mitogenomic sequences of the species belonging to the order Myxiniformes were retrieved from GenBank in NCBI (http://www.ncbi.nlm.nih.gov/). They were aligned together with that of *M. fernholmi* in this study and refined manually. For the phylogenetic analysis, the nucleotide matrix of 12 protein-coding genes, excluding *nad6* was created and divided into three partitions, consisting of 2998 bp each for the first, second and third bases of each codon triplet, respectively. A phylogenetic tree was reconstructed using RAxML 7.0.4 (Stamatakis 2006) for maximum-likelihood (ML) analysis.

The mitogenome of *M. fernholmi* was a circular molecule of 18,862 bp in total length, consisting of 13 protein-coding genes, two ribosomal RNA genes, and 22 transfer RNA genes, of which gene content is identical not only to that of the congeneric *Myxine glutinosa* (Delarbre et al. 2001) but also to those of typical vertebrates. Its gene order was also identical to those of typical vertebrates.

With the full-length mitogenomic sequence of *M. fernholmi*, a phylogenetic tree was reconstructed by the ML method, using the nucleotide sequence matrix from 12 protein-coding genes (Figure 1). In the resulting tree, all hagfish consistently formed the monophyletic group with 100% bootstrap value with respect to the two lamprey outgroups. Among the lineage, two *Eptatretus* species belonging to the subfamily Eptatretinae were consistently separated from two *Myxine* species belonging to the subfamily Myxininae. *Myxine fernholmi* in this study clustered with the congeneric *Myxine glutinosa* with 51% bootstrap value.

**Figure 1.**
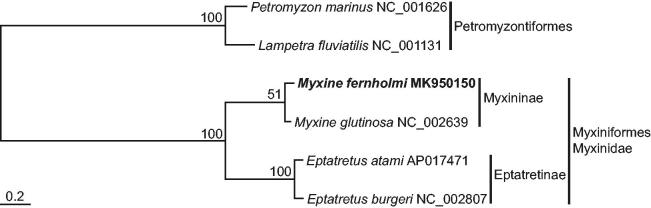
Maximum-likelihood (ML) phylogeny based on the full-length mitochondrial genomes from the hagfish belonging to the order Myxiniformes. The nucleotide sequence matrix included the three codon positions of the 12 protein-coding genes. A bootstrap value above 50% in the ML analysis is indicated at each node. *Myxine fernholmi* analyzed in this study is shown in bold.

In this study, the complete mitogenome of *M. fernholmi* was analyzed for the first time, and the phylogenetic tree among hagfish was reconstructed with reference to their mitogenomic data. Our data will provide baseline data for the molecular identification, geographical distribution, population genetics and conservation biology for the rare natural living resource, *M. fernholmi* in the Southwest Atlantic Ocean.
